# Hepatobiliary Disease Resection in Patients with Advanced Epithelial Ovarian Cancer: Prognostic Role and Optimal Cytoreduction

**DOI:** 10.1245/s10434-020-08989-3

**Published:** 2020-08-10

**Authors:** Violante Di Donato, Andrea Giannini, Ottavia D’Oria, Michele Carlo Schiavi, Anna Di Pinto, Margherita Fischetti, Francesca Lecce, Giorgia Perniola, Francesco Battaglia, Pasquale Berloco, Ludovico Muzii, Pierluigi Benedetti Panici

**Affiliations:** 1 Department of Maternal and Child Health and Urological Sciences, “Sapienza” University of Rome, Umberto I Hospital, Rome, Italy; 2 Department Obstetrics and Gynecological Hospital Santa Maria Goretti of Latina, “Sapienza” University of Rome, Rome, Italy; 3 Department of General Surgery and Organ Transplantation, “Sapienza” University of Rome, Rome, Italy

## Abstract

**Objective:**

The purpose of this study was to evaluate the feasibility and safety in terms of prognostic significance and perioperative morbidity and mortality of cytoreduction in patients affected by advance ovarian cancer and hepato-biliary metastasis.

**Methods:**

Patients with a least one hepatobiliary metastasis who have undergone surgical treatment with curative intent of were considered for the study. Perioperative complications were evaluated and graded with Accordion severity Classification. Five-year PFS and OS were estimated using the Kaplan–Meier curve.

**Results:**

Sixty-seven (20.9%) patients had at least one metastasis to the liver, biliary tract, or porta hepatis. Forty-four (65.7%) and 23 (34.3%) patients underwent respectively high and intermediate complexity surgery according. Complete cytoreduction was achieved in 48 (71.6%) patients with hepato-biliary disease. In two patients (2.9%) severe complications related to hepatobiliary surgery were reported. The median PFS for the patients with hepato-biliary involvement (RT = 0 vs. RT > 0) was 19 months [95% confidence interval (CI) 16.2–21.8] and 8 months (95% CI 6.1–9.9). The median OS for the patients with hepato-biliary involvement (RT = 0 vs. RT > 0) 45 months (95% CI 21.2–68.8 months) and 23 months (95% CI 13.9–32.03).

**Conclusions:**

Hepatobiliary involvement is often associated with high tumor load and could require high complex multivisceral surgery. In selected patients complete cytoreduction could offer survival benefits. Morbidity related to hepatobiliary procedures is acceptable. Careful evaluation of patients and multidisciplinary approach in referral centers is mandatory.

**Electronic supplementary material:**

The online version of this article (10.1245/s10434-020-08989-3) contains supplementary material, which is available to authorized users.

Epithelial ovarian cancer (EOC) is the leading cause of death from gynecologic malignancy.^1^-^3^ In 2019, an estimated 22,530 women will be diagnosed in the United States, and approximately 13,980 of these women will die from this disease.^4^ Due to the lack of early screening, many women are diagnosed with ovarian cancer when it is already at stages III or IV,^5^,^6^ and they are treated by debulking surgery and platinum-based chemotherapy. Several studies confirmed improved survival in patients who have complete resection (CR) defined as no visible residual disease.^7^,^8^

Increase tumor load is associated with increase of surgical procedures required to obtain no residual tumor. In the past year, the presence of hepato-biliary metastasis required extensive upper abdominal procedures was associated with low chance of optimal cytoreduction.^7^-^9^ However, increase perioperative care and gynecology’s surgical skills have achieved better results in terms of optimal cytoreduction also in presence of disease in challenging area. Initial experiences in liver resection, biliary surgery, and porta hepatis have been reported.^8^,^10^-^12^ The purpose of this study was to evaluate the feasibility and safety in term of prognostic impact and perioperative morbidity of cytoreduction in patients affected by advance ovarian cancer and at least one histologically proven hepatobiliary metastasis.

## Materials and Methods

The medical records of all patients elected to surgical intervention for Ovarian Cancer at the Maternal and Child and Urological Sciences Department of Umberto I Hospital, Sapienza, University of Rome, from January 2005 to December 2018, were retrospectively extracted from a prospective collected database. Inclusion criteria were: histologically confirmed ovarian cancer, informed consent signed, follow-up completed, and at least one histologically proven hepatobiliary metastasis. Exclusion criteria were as follows: patients who denied access to their medical information for research purposes, ovarian cancer not histologically confirmed, lost follow-up, and synchronous invasive cancers. All patients signed informed consent to the processing of personal data. The study received the ethical approval of our Department Review Board. Demographic data, operative and pathological reports, and clinical outcomes were extracted from the database. Missing data and data concerning preexisting medical conditions present at the time of primary surgery were extracted from the patient’s medical records. The following preoperative information were collected for statistical analysis: age, body mass index (BMI), serum level of Ca125, hemoglobin (Hb), and albumin. Intraoperative information was recorded, including operative time, surgical procedures performed, and diameter of largest residual tumor nodule. Information obtained from the final pathology report included stage, tumor histology, and tumor grade. All patients were staged according to the International Federation of Gynecology and Obstetrics (FIGO) system. Surgical complications were graded according to Accordion severity Classification of postoperative complication expanded classification, divided in early (< 30 days) and late (> 30 and < 60 days).^13^ The complexity of the surgical procedures performed was evaluated using the Surgical Complexity Scoring System (SCS) from 1 to 3 (simple to complex, respectively).^14^ Surgical outcomes in ovarian cancer are classified according to the size of the largest residual tumor present after surgery, which is one of the most important prognostic factors; resection of the tumor is considered as complete if no macroscopically visible tumor remains.^15^

The primary outcome of this study was to evaluate the prognostic impact in terms of progression disease-free survival (PFS) and overall survival (OS) of primary optimal cytoreduction in patients affected by advance ovarian cancer and at least one hepatobiliary metastasis. The secondary endpoint was to evaluate perioperative morbidity.

### Statistical Analysis

Standard statistical analysis was used to evaluate descriptive analysis, such as mean, frequencies, and percentages. Incidence of event was analyzed for statistical significance by using the Fisher exact test. Normality testing (D’Agostino and Pearson test) was performed to determine whether data were sampled from a Gaussian distribution. The *t* test and Mann–Whitney *U* test were used to compare continuous parametric and nonparametric values, respectively. Predicting variables were evaluated for their association with complication rate on the basis of logistic regression model. Odds ratio (OR) and 95% confidence intervals (CIs) were calculated for each comparison. PFS was calculated in months from the date of surgery to the last follow-up or date of first recurrence. OS was calculated in months from the date of surgery to the last follow-up or date of death from disease. Five-year PFS, and OS were estimated using the Kaplan–Meier curve. Cox regression analysis was used to calculate both univariate and multivariate analysis for variables influencing PFS and OS. Associations were summarized by calculating hazard ratio (HR) and corresponding 95% CI. All calculated *p* values were two-sided. The *p* value < 0.05 were considered statistically significant. Statistical analysis was performed with IBM Microsoft’s SPSS Statistics version 25.0 for Mac (IBM Corp., Armonk, NY).

## Results

From January 2005 to December 2018, 320 patients were surgically treated for Advanced Ovarian Cancer and evaluated for this study. The median follow-up was 36 months. Sixty-seven (20.9%) patients had at least one liver, biliary metastasis, or porta hepatis and celiac nodes involvement. Table 1 shows the patients’ characteristics. Mean age was 53.80 ± 11.58 years, and mean body mass index (BMI) was 24.57 ± 4.26. In particular, 55 patients (82.1%) had a hepatic lesion, 20 (29.8%) had biliary lesion, 3 (4.5%) had portal vein nodes, 3 (4.5%) had involvement of common hepatic artery, and 6 (8.9%) had involvement of celiac nodes (Table 2). Forty-five (67.1%) patients were diagnosed at FIGO stage IIIC and 22 (32.9%) at IV. The most represented histology was serous papilliferous ovarian cancer (82%). Mean preoperative Ca 125 was 687.52 ± 100.8 UI/ml. Forty-one (61.2%) patients underwent PDS surgery, while 26 (38.8%) underwent surgery after neoadjuvant chemotherapy. Mean surgical time was 297 ± 110 min, and median inpatient day was 8 (range 5–22). Table 3 reports procedures associated. Twenty-seven patients (40.2%) had a diaphragmatic surgery, 2 (3.0%) gastric surgery, 16 (23.9%) pancreatic surgery, 20 (29.9%) splenic surgery, 23 (34.3%) rectal surgery, 33 (49.3%) ileum surgery, and 16 (23.9%) large bowel surgery. In addition, 6 patients (9.0%) had a pelvic lymphadenectomy, 10 (14.9%) para-aortic lymphadenectomy, and in 4 (5.9%) pelvic and paraaortic lymphadenectomy for presence of bulky nodes. No patients received low complexity surgery, whereas 65.7% and 34.3% of patients underwent respectively high and intermediate complexity surgery according to Aletti et al.^14^. Complete cytoreduction was achieved in 48 (71.6%) patients with hepatobiliary disease, while in 19 patients (28.4%) residual tumor was present. Compared with the overall population, the rate of complete cytoreduction was statistically lower in patients with hepatobiliary metastasis (*p* = 0.0092). In particular, residual tumor was < 1 cm in 13 patients (19.4%) and > 1 cm in 6 (8.9%). Of 19 patients who not achieved complete resection, 8 patients (42.1%) had mesenteric involvement with superior mesenteric artery infiltration, 5 patients (26.3%) had small-bowel diffuse carcinosis, in 2 patients (10.5%) hepatic artery was infiltrated, 2 patients (10.5%) had common bile duct infiltration, 1 patient (5.3%) portal vein massive involvement, and 1 patient (5.3%) multiple parenchymal hepatic metastasis (Table S1). Thirty-nine (58.2%) patients had at least one postoperative complication. Seven patients (10.4%) had severe complications (G3–G5): particularly, two cases of abdominal abscesses that required postoperative draining; two cases of pancreatitis treated with drainage position, and one case of dehiscence of rectal anastomosis requiring reintervention. In two patients (2.9%), the severe complications reported were directly related to hepatobiliary surgery. A patient underwent large diaphragmatic and hepatic resection; the complete mobilization of the liver has enabled trans-diaphragmatic herniation 4 days after surgery. She underwent laparotomy with hernia reduction and diaphragmatic reparation with Prolene. A patient of our series was readmitted 10 days after wedge liver resection for occurrence of hepatobiloma. In that patient, minor bile leakage was secondary to partial ischemic necrosis and open abdominal reoperation was required.

**Table 1:** Clinic and characteristics of patients with hepatobiliary disease (*n* = 67)

Age (mean ± SD)	53.80 ± 11.58
BMI (mean ± SD)	24.57 ± 4.26
Hystotipe	
Serous papillary	55 (82%)
Endometrioid	3 (4.5%)
Clear cell	4 (6%)
Other	5 (7.5%)
Grading	
G1	2 (3%)
G2	14 (20.9%)
G3	51 (76.1%)
FIGO stage	
IIIC	45 (67.1%)
IV	22 (32.9%)
Preoperative albumine (g/dL)	4.2 (2.2–5.7)
Preoperative Hemoglobin (g/dL)	12.7 ± 1.19
Preoperative Ca 125 (U/ml)	687.52 ± 100.8
Surgery	
PDS	41 (61.2%)
IDS	26 (38.8%)
Inpatients day	8 (5–22)
Operative time	297 ± 110

*SD* standard deviation; *BMI* body mass index; *PDS* primary debulking surgery; *IDS* interval debulking surgery

**Table 2:** Site of disease

Hepatic	55 (82.1%)
Intraparenchymal	20 (29.8%)
Glisson’s capsule	35 (52.3%)
Biliary tract	20 (29.8%)
Celiac lymph node	6 (8.9%)
Common hepatic arteries	3 (4.5%)
Portal vein	3 (4.5%)

**Table 3:** Surgery procedures

Hepatobiliary procedures	
Hepatic surgery	
Liver resection	20 (29.8%)
Glisson resection	35 (52.3%)
Biliary surgery	
Biliary tract	20 (29.8%)
Celiac lymphadenectomy	6 (8.9%)
Hepatic artery	3 (4.5%)
Portal vein nodes	3 (4.5%)
Associated procedures	
Diaphragmatic surgery	
Peritoneal	18 (26.5%)
Resection	9 (1.2%)
Pancreatectomy	16 (23.9%)
Splenic surgery	20 (29.4%)
Gastric surgery	2 (2.9%)
Lymphadenectomy	
Pelvic	6 (8.8%)
Para-aortic	10 (14.7%)
Pelvic + para-aortic	4 (5.9%)
Rectal surgery	23 (33.8%)
Ileum surgery	33 (48.5%)
Large bowel surgery	16 (23.5%)

**Fig. 1:**
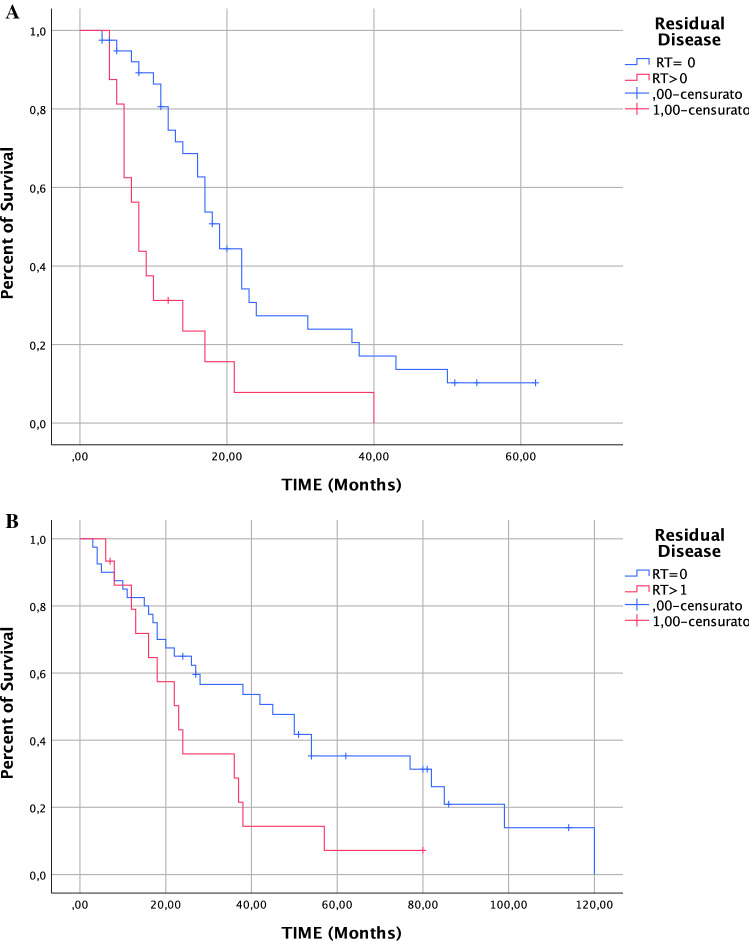
**a** Progression-free survival (PFS) of patients with Residual Disease 0 (blue line) and Residual Disease more than 0 (red line). **b** Overall survival (OS) of patients with Residual Disease 0 (blue line) and Residual Disease more than 0 (red line)

The vast majority (76.9%, 30/39) of complications was mild (G1–G2). Rate of complications according to surgical complexity is showed in Table 4. Two patients (2.9%) died within 90 days from surgery: one patient for pulmonary embolism, and one for sepsis and multiorgan dysfunction syndrome (MODS). The median PFS for stage III/IV ovarian cancer patients with hepatobiliary involvement was shorter compared with patients without hepatobiliary involvement (17 months; 95% CI 13.6–20.4 vs. 19 months 95% CI 14.2–23.8; *p*: 0.016) (not part of the present study). The median PFS for the patients with hepatobiliary involvement comparing RT = 0 versus RT > 0 was 19 months (95% CI 16.2–21.7 months) versus 8 months (95% CI 4.1–11.9), *p*: 0.001 (Fig. 1a). The median PFS for patients who achieved complete cytoreduction (RT = 0) with or without hepatobiliary involvement (not part of the present study) was not statistically different: 19 (95% CI 16.2–21.7) versus 23 (95% CI 13.9–32.0) (*p*: 0.24). In patients with residual tumor after surgery (RT > 0), the median PFS was 8 months regardless of hepatobiliary involvement (*p*: 0.64).The median OS for the patients with hepatobiliary involvement who achieved complete cytoreduction (RT = 0) was 45 months (95% CI 21.2–68.8 months) and for patients with RT > 0 was 23 months (95% CI 13.9–32.0; Fig. 1b).

**Table 4:** Rate of complications according to surgical complexity

Accordion score	Total (*n* = 67)	High complexity (*n* = 44)	Intermediate complexity (*n* = 23)
0 (No complication)	28 (41.8%)	13 (29.5%)	15 (65.2%)
Mild complication (G1–G2)	30 (44.8%)	22 (50%)	8 (34.8%)
Severe complication (G3–G5)	7 (10.4%)	7 (15.9%)	0
90-Day mortality	2 (2.98%)	2 (4.5%)	0

## Discussion

One of five patients affected by advanced stage ovarian cancer had hepatobiliary involvement in our series. These data are consistent with literature, despite only few authors investigated this aspect.^12^,^15^-^17^ In autoptic analysis half of the patients with ovarian cancer have liver metastases.^18^ Computed tomography (CT) scan failed to identify HCLN or porta hepatitis involvement in almost all cases.^19^ Instead, the positron emission tomography (PET) CT scan seems to have a superior diagnostic sensibility.^12^ Therefore, real incidence of hepatobiliary involvement could be underestimated in published series. As a consequence, diaphragms after liver mobilization, epiploon retro cavity, or porta hepatis should be always explored during cytoreductive surgery to identify potentially nonvisible disease at preoperative instrumental examinations.

One of the major finding of the present study was the rate of 71.6% of patients affected by ovarian cancer with hepatobiliary involvement who had macroscopically complete resection of all visible tumor. Our data seem to be consistent with literature (Table 5) even if population of studies are heterogeneous.^6^,^12^,^15^-^17^,^19^-^29^ As reported in a recently published multicentric prospective trial, 8.5% (55/647) and 20% (130/647) of selected ovarian cancer patients underwent partial hepatectomy and resection of porta hepatis, achieving a complete debulking.^15^ This study suggested that an optimal surgery also could be achieved even though liver and porta hepatis are involved. Moreover, the reason of incomplete cytoreduction was in 42% (8/19) of cases was not correlated with residual disease in hepatobiliary area. This finding is of really importance since some authors have classically considered the presence of a hepatobiliary metastasis as a criterion of unresectability. In our opinion, nonresectability is even more related to the spread of disease than to the presence of a single hepatobiliary metastasis. Nevertheless, excluding patients only on account of presence of a hepatobiliary metastasis could deprive a considerable number of women the benefits of surgery, particularly those with an acceptable performance and nutritional status, in whom gross disease can reasonably be removed. Although, often, but not always, hepatobiliary involvement may be related to a higher spread of disease. Consistently, Rodriguez et al. found that patients requiring upper abdominal procedures due to disease spread at hepatobiliary location had higher preoperative disease overall volume compared with patients who did not require upper abdominal procedures.^24^ Because the majority of patients with hepatobiliary metastasis had a high tumor burden, they therefore required multiple complex procedures. Our results confirm these findings; indeed no patients in our series received low complexity surgery, whereas two of three patients underwent high complexity surgery. Anyway, not all women should be candidates to these highly complex surgical procedures, but only those who may reach survival benefit from optimal residual tumor. Treatment must be personalized taking into account not only tumor spread but also patient’s characteristics. The challenge of the decision-making process for high-risk patients for surgery with curative intent versus palliative treatment lies in the balance between an expected improved survival, if complete debulking is achieved and the expected surgical morbidity and mortality. In our series 58% of patients with metastatic involvement of hepatobiliary region had at least one complication, but only in 10.4% of cases were severe. Because the study includes only patients undergone surgical treatment with curative intent and at least one histologically proven hepatobiliary metastasis, the worst patients have been considered more frequently inoperable or unresectable. This carefully perioperative assessment could explain the low severe postoperative morbidity experienced. Moreover, complications seem to be significantly associated with high scores of Surgical Complexity Score, and in only two patients (2.9%), the severe complications reported were directly related to hepatobiliary surgery.

**Table 5:** Main features of studies investigating the clinical role of liver and PH resection in ovarian cancer patients

References	No. patients	Stage of disease	Liver resection, n (%)	PH resection, n (%)	No RT	No RT in liver and PH area	Morbidity (classification)	Morbidity related to LIVER or PH (classification)
Bristow et al.^16^	84 Primary	IV 84 (100%)	37 (44%)	–	30% RT < 1	16% RT < 1	Overall: 32% Mortality: 6%	–
Chi^a^ et al.^20^	70 Primary	III 59 (84%) IV 11 (16%)	3 (4.3%)	3 (4.3%)	76% RT < 1	–	G1–2: 50% G3–5: 6% Mortality: 0.7% MSKCC	–
Eisenhauer et al.^21^	57 Primary	III 41 (72%) IV 16 (28%)	9 (16%)	8 (14%)	13 (23%)	–	Overall 7 (12%)	–
Chi et al.^22^	141 Primary	III 103 (73%) IV 38 (27%)	18 (13%)	14 (10%)	42 (30%)	–	G3–5 31 (22%) Mortality: 1.4%	–
Song et al.^23^	101 Primary 54 Recurrence	–	6 (3.9%)	Primary 2 (1.9%) Recurrence: 9 (16.7%)	144 (100%)	10 (90.9%)	Any G: 4 (4%) Mortality: 0%	0%
Martinez et al.^17^	20 Primary 8 Recurrence	III 22 (78.5%) IV 6 (21.5%)	7 (25%)	2 (7.1%)	27 (96.4%)	–	–	G3–5 10 (35.7%) Mortality: 1 (3.6%) CTCAE, 2009
Raspagliesi et al.^19^	37 Primary	III 29 (78.3%) IV 8 (21.7%)	3 (8.1%)	4 (10.8%)	34 (91.9%)	–	G1–3 8 (21.6%) Mortality 0% CTCAE, 2010	–
Rodriguez et al.^24^	482 Primary	III 392 (81.3%) IV 90 (18.7%)	112 (23.2%)	7 (0.2%)	141 (29.3%)	–	–	–
Martinez et al.^25^	34^b^ Primary 7^b^ Recurrence	III 32 (78%) IV 9 (22%)	–	41 (100%)^b^	40 (97.6%)	–	–	–
Benedetti Panici et al.^26^	121 Primary	III 96 (79.4%) IV 25 (20.6%)	48 (40%)	24 (19.8%)^c^	91 (75.1%)	–	G1–2 22 (17.8%) G3–4 23 (19%) Mortality: 0.7% CTCAE, 2009	–
Tozzi et al.^12^	31 Primary	III 24 (77.4%) IV 7 (22.6%)	–	31 (14.3%)^b^	–	28 (90.3%) $	G3: 3 (23.1) Mortality 0% Clavien-Dindo	–
Gallotta et al.^27^	85 Primary	III 77 (90.6%) IV 8 (9.4%)	8 (9.4%)	–	73 (85.9%)	–	Overall: 58 (68.2%) G3–G4: 30(51.7%) Mortality: 3.5% MSKCC	–
Harter et al.^15^	647 Primary	I to IIa 32 (4.9%) IIb to IIIa 93 (14.4%) IIIb to IV 505 (77.7%)	55 (8.5%)	130 (20.1%)^d^	643 (99.4%)^e^	–	Overall: 495 (76.5%) Mortality: 2%	.
Angeles et al.^28^	43 Primary	III 36 (83.7%) IV 7 (16.3%)	8 (18.6%)	22 (51.2%)	43 (100%)	43 (100%)	G3–5: 13 (30.2%) Mortality 0% Clavien-Dindo	0%

*PH* porta hepatis; *RT* residual tumor; *MSKCC* Memorial Sloan Kettering Cancer Center; *CTCAE* Common terminology criteria for adverse events

^a^January 2001 to May 2002

^b^Celiac nodes included

^c^Biliary tract included

^d^Lesser omentum included

^e^Patients underwent randomization only if macroscopically complete resection had been achieved

Hepatobiloma is a potential complication in liver surgery^30^ usually treated with percutaneous catheter drainage. In order to prevent this type of complications, some authors suggested the use of intraoperatory ultrasound to evaluate rapport between liver metastasis and bile ducts.^31^ In liver surgery, it is important not only to avoid directly injure but also to avoid burning and necrotizing the intrahepatic bile vessels. In liver surgery, it is important not only to avoid directly injure but also to avoid burning and necrotizing the intrahepatic bile vessels. Current data are not available to allow accurate risk prediction of surgical mortality in ovarian cancer. Different algorithms have been proposed, but nowadays there is not unanimous consensus in literature.^32^ A recent metanalysis based on 46 studies that involved 18,579 patients has evaluated predictors of 30-day mortality in patients undergoing primary cytoreduction for ovarian cancer demonstrating that combined effects of increased age and advanced clinical stage factors greatly increased the risk of perioperative mortality.^32^,^33^ However, some authors have showed benefits of complete cytoreduction in terms of survival, regardless of age.^33^,^34^ As reported by Langstraat et al., despite elderly patients had increase risk surgical morbidity and mortality after multivisceral surgery, they also could potentially have survival advantage from radical surgical approach similar to that of younger one.^35^,^36^ However, patients’ comorbidities seem to have a strongly effect on mortality and morbidity, and aging-associated state of increased vulnerability, in frail patients, influences complications rate and survival.^37^ Our study suggests that the presence of metastatic involvement of hepatobiliary region represents a negative indicator of survival (hepatobiliary vs. no hepatobiliary involvement OS 28 vs. 46 months, *p*: 0.03; PFS 17 vs. 19 months, *p*: 0.03); however, the removal of all visible tumor remains an important prognostic factor impacting on the OS (RT = 0 vs. > 0; 45 vs. 23 months), and PFS (RT = 0 vs. > 0; 19 vs. 8 months, *p*: 0.001).

## Conclusions

Hepatobiliary involvement in patients with EOC often is associated with high tumor load and could be considered as independent risk factor for early recurrent disease. The surgical treatment of EOC is a complex issue and requires a multidisciplinary assessment. Tailored strategy and multivisceral surgical approach, in selected patients, could allow to achieve complete resection of disease. Demolitive surgery should be limited to cases in which optimal residual tumor can be achieved, but selection is a challenge. All patients with EOC should be referred to specialized dedicated center in order to receive the best treatment. Multicentric prospective studies to confirm our results are warranted.

## Electronic supplementary material

Below is the link to the electronic supplementary material.

Supplementary material 1 (DOCX 13 kb)
