# Supplementary material for: Hepatobiliary Disease Resection in Patients with Advanced Epithelial Ovarian Cancer: Prognostic Role and Optimal Cytoreduction
Source: Ann Surg Oncol. 2020 Aug 10;28(1):222–30. doi: 10.1245/s10434-020-08989-3 (PMC7752869; doi:10.1245/s10434-020-08989-3)
Supplement: Supplementary file 1 — Supplementary material 1 (DOCX 13 kb) [file 10434_2020_8989_MOESM1_ESM.docx]

**Table S1. Causes of non-optimal cytoreduction (n=19)**

| Superior mesenteric artery involvement | 8/19 (42,1%) |
| --- | --- |
| Small bowel disease | 5/19 (26,3%) |
| Hepatic artery involvement | 2/19 (10.5%) |
| Common bile duct involvement | 2/19 (10.5%) |
| Portal vein involvement | 1/19 (5.3%) |
| Hepatic parenchymal involvement | 1/19 (5.3%) |
